# Interdisciplinary Collaboration to Teach about End-of-Life Issues and Advance Care Planning

**DOI:** 10.1093/geroni/igaf122.410

**Published:** 2025-12-31

**Authors:** Tina Newsham, Alissa Dark-Freudeman, Elizabeth Fugate-Whitlock, Jamy Chulak

**Affiliations:** The University of North Carolina at Wilmington, Wilmington, North Carolina, United States; The University of North Carolina at Wilmington, Wilmington, North Carolina, United States; The University of North Carolina at Wilmington, Wilmington, North Carolina, United States; The University of North Carolina at Wilmington, Wilmington, North Carolina, United States

## Abstract

Topics related to end-of-life (EOL) and advance care planning (ACP) can be challenging as they may activate negative assumptions and experiences for students and instructors. Further complicating teaching about these topics, they can (and, we argue, should) be addressed through multiple disciplinary lenses, and ideally in an interdisciplinary fashion. In this paper, we share an interdisciplinary effort to teach about EOL and ACP at the University of North Carolina Wilmington. Faculty in Psychology, Gerontology, Philosophy, and Respiratory Therapy collaborated to create an interdisciplinary module that was integrated into one class from each participating discipline. Students were grouped across disciplines and worked together to create content for a Virtually Inclusive Patient online module that can be used in a variety of courses. Students completed guided reflections regarding what they learned about and from students with different disciplinary perspectives and how the interdisciplinary collaboration resulted in outcomes that would otherwise have been absent in a singular-discipline effort. We highlight here the interdisciplinary nature of the collaboration between the faculty leading this effort as well as that of the students participating in this project.

